# Analysis of Phenolic Content in Grape Seeds and Skins by Means of a Bio-Electronic Tongue

**DOI:** 10.3390/s20154176

**Published:** 2020-07-27

**Authors:** Cristina Garcia-Cabezon, Guilherme Gobbi Teixeira, Luís Guimaraes Dias, Coral Salvo-Comino, Celia García-Hernandez, Maria Luz Rodriguez-Mendez, Fernando Martin-Pedrosa

**Affiliations:** 1 Department of Materials Science, Universidad de Valladolid, 47011 Valladolid, Spain; fmp@eii.uva.es; 2 Centro de Investigação de Montanha (CIMO), ESA, Instituto Politécnico de Bragança, 5301 Bragança, Portugal; guilhermegob@gmail.com (G.G.T.); ldias@ipb.pt (L.G.D.); 3 Group UVaSens, Escuela de Ingenierías Industriales, Universidad de Valladolid, Paseo del Cauce, 59, 47011 Valladolid, Spain; coraldeugena@hotmail.com (C.S.-C.); celiagarciahernandez@gmail.com (C.G.-H.)

**Keywords:** electrochemical sensor, bio-electronic tongue, grape seed, grape skin, phenol

## Abstract

A bio-electronic tongue has been developed to evaluate the phenolic content of grape residues (seeds and skins) in a fast and easy way with industrial use in mind. A voltammetric electronic tongue has been designed based on carbon resin electrodes modified with tyrosinase combined with electron mediators. The presence of the phenoloxydase promotes the selectivity and specificity towards phenols. The results of multivariate analysis allowed discriminating seeds and skins according to their polyphenolic content. Partial least squares (PLS) has been used to establish regression models with parameters related to phenolic content measured by spectroscopic methods i.e., total poliphenol content (TPC) and Folin–Ciocalteu (FC) indexes. It has been shown that electronic tongue can be successfully used to predict parameters of interest with high correlation coefficients (higher than 0.99 in both calibration and prediction) and low residual errors. These values can even be improved using genetic algorithms for multivalent analysis. In this way, a fast and simple tool is available for the evaluation of these values. This advantage may be due to the fact that the electrochemical signals are directly related to the phenolic content.

## 1. Introduction

In grape berries, polyphenolic compounds are mainly found in skins and seeds. Many factors are essential in the phenolic composition of grapes, namely degree of ripeness, climate conditions, grapevine variety and berry size [1,2]. The most abundant phenolic compounds in grape skins are flavonols, while grape seeds have high levels of flavan-3-ols [2]. During wine making, these compounds are transferred to wines and have an important influence in the final organoleptic characteristics of wines.

The grape marcs (seeds and skin remains of grapes after pressing) have a high content on polyphenols, increasing the interest of exploiting these sub-products [3]. For instance, Bekhit et al. [4] showed that extracts obtained from wine production residues may have anti-influenza virus activity. Besides, some grape pomaces have shown antioxidant activity [5]. In summary, reduction of negative costs, as well as improvement if the sustainability in wine making are the positive incomes when its bio-products residues turn into useful technologies [6,7].

The total extractable phenolics are found mainly in the seeds (60–70%), then in the skin (28–35%), and finally in the pulp (only about 10%) [8]. Therefore, the extraction technique is an important issue when focusing on isolating, identifying and using these compounds. There is no standard method to do so. Solid–liquid extraction using organic solvents as ethanol has shown good efficiency [9,10].

Spectrophotometry is one of the methods used to determine the phenolic content of grape samples [3,4,5]. Other techniques commonly used for this purpose are colorimetry [6] HPLC (high performance liquid chromatography) [1,11], Raman and ATR–FTIR (attenuated total reflection—Fourier transform infra-red) [12].

In the last years, electrochemical sensors were improved in order to give us rapid, simple, cheap and sensitive information about the polyphenolic content [13,14]. Modified carbon electrodes have been tested recently as voltammetric sensors for the analysis of phenolic compounds, especially when modified with nanomaterials [15,16,17,18]. The electrocatalytic properties—stability and large surface area of metal oxide nanoparticles—make them interesting materials to fabricate electrochemical sensors dedicated to the analysis of antioxidants [19,20]. Recently, nickel oxide nanoparticles (NiONPs) were successfully used in monitoring the phenolic maturity of red grapes [21]. Other materials have demonstrated to be excellent modifiers in sensors dedicated to the detection of phenols. One excellent example is the lutetium bisphthalocyanine (LuPc_2_), a sandwich-type derivative with free radical character, which is an intrinsic semiconductor that shows excellent electrocatalytic activity towards phenols [22,23,24,25].

The specificity of electrochemical sensors can be improved by modifying the electrode surface with an enzyme. Enzyme-based electrochemical sensors based on phenoloxidases such a tyrosinase (Tyr) can exhibit a faster response, enhanced operational repeatability, lower background current, lower limit of detection and higher sensitivity [26,27,28,29]. The interaction between tyrosinase and electron mediators, such as nanoparticles or phthalocyanines, can improve sensor operation [29].

In spite of the excellent performance shown by electrochemical sensors, their use in complex matrixes is limited due to the presence of interferences. This problem can be overcome using the so-called electronic tongues, where an array of sensors with cross-selectivity is coupled to a pattern recognition software [30,31]. Amperometric and voltammetric sensor arrays have widely used to analyze wines and musts [13,32,33]. Electronic tongues provide global information about the sample instead of information about specific components. More recently, bioelectrochemical sensors were successfully included in sensor arrays to form bio-electronic tongues. The presence of biosensors can help to add specificity towards different compounds. This means that bio-electronics tongues combine the advantages of classical electronic tongues with the typical specificity of biosensors. Bio-electronic tongues have been used to analyze wines or musts [34,35], however they have not been applied to the analysis of wine making marcs.

In order to process the data obtained from the electrode array, multivariate statistical studies are required given that the signal from the sensors contain meaningful information of the samples. It is preferred to use pattern recognition techniques that include partial least squares (PLS) [36] to distinguish samples by their organoleptic characteristics and to establish a relationship between the responses of the sensors and the characteristics of the sample as the polyphenol content [13,36,37]. A method for selecting variables called genetic algorithm in partial least squares (GA–PLS) has also been used, which is one of the most widely used techniques for the selection of variables and for improving the performance of PLS [38,39].

The main objective of this work was to develop a multisensory system (a bio-electronic tongue, ET) based on carbon electrodes modified with sensing nanomaterials and enzymes to evaluate the polyphenol content of wine making residues (seeds and skin), obtained from eight grape varieties used for wine making in the region of Castilla y León, Spain, valuing this kind of industrial waste.

## 2. Materials and Methods

### 2.1. Materials and Working Electrodes Preparation

Catechol (>99.0%), nickel (II) oxide nanoparticles (≥99.8%, <50 nm particle size), ethanol (≥99.8% HPLC grade), tyrosinase (Tyr from Agaricus bisporus activity of 1000 U mg^−1^, CAS 9002-10-2), sodium phosphate monobasic and dibasic (≥99.0%), potassium chloride (≥99.0%) and Araldite^®^ resin were obtained from Sigma–Aldrich (France). Glutaraldehyde (50% aqueous solution) was purchased from Alfa Aesar (Haverhill, MA, USA). The lutetium (III) bisphthalocyaninate (LuPc_2_, 0.05 g L^−1^) was synthesized using a method developed by our group [40]. Deionized water from MilliQ (resistivity 18.2 MΩ·cm) was used in all experiments.

Working graphite composite electrodes were prepared based on the mixture of graphite with epoxy resin Araldite and with/without the addition of substances to modify the electrodes’ electrocatalytic properties in order to verify the electrodes performance, together with the tyrosinase enzyme, in the analysis of grape seeds and skins samples. Thus, nickel oxide nanoparticles (NiO NPs) and lutetium phthalocyanine (LuPc_2_) were chosen to modify composition of the graphite composite electrodes (electrocatalytic modifying compounds, EM). The electrode array was composed of six working electrodes; the first three electrodes were: C (carbon + Araldite^®^ resin; 50–50%); C-NiONPs (carbon + Araldite^®^ resin + NiO NPs; 49.5–49.5-1%, respectively); C-LuPc_2_ (carbon + Araldite^®^ resin + LuPc_2_; 49–49-2%, respectively).

The remaining three electrodes were replicas of the first but with the surface modified with the addition of the tyrosinase enzyme: C-Tyr, C-NiONPs-Tyr and C-LuPc_2_-Tyr. Tyrosinase was immobilized by drop-casting 20 µL of a tyrosinase solution (5 mg in 1 mL of buffer 0.01 M) twice in the electrode’s surface. Afterward, the electrode was immersed in a buffer phosphate solution (pH 7; 0.01 M-0.5393 g of NaH_2_PO_4_ and 0.7318 g of Na_2_HPO_4_ in 1000 mL) for 1 min. After drying, it was left in contact with vapors of glutaraldehyde (50%) for 20 min and again 30 s in the buffer solution, being finally stored at 4 °C until the analysis.

### 2.2. Samples

The wine making residues samples were obtained from the vineyards “Bodega Cooperativa de Cigales” and “Instituto Tecnológico Agrario de Castilla y León (ITACYL)”, both located in the Valladolid area of Castilla y León, in Spain. This collection is from the 2015 grape harvest and is composed of eight varieties of red grapes, namely Juan García (J), Mencia Regadio (MR), Mencia Secano (MS), Rufete (R), Prieto Picudo (P), Garnacha (G), Tempranillo (T) and Cabernet (C).

In order to test them electrochemically, the samples of wine making residues (seed and skin grape) were separately dried at 60 °C for 24 h and, after grinding and sifting, particles smaller than 400 µm were again dried at 60 °C for 2 h. The extraction process was made by mixing at a rotation speed of 1200 rpm, 1 g of each dried sample with 40 mL of 50% ethanol solution for 2 h, in ambient temperature. After that, the extracts were centrifuged (1200 rpm) for 10 min and the supernatant was used as the final product.

The phenolic content of the grape seeds and skins was determined by using two spectrophotometric analytical methods: total polyphenolic content by measuring absorbance at 280 nm 280 nm (TPC index) and total phenolic content by using the Folin–Ciocalteu method (FC index) by measuring the absorbance at 750 nm. [41].

### 2.3. Voltammetric Characterization and Tests

In order to make an electrochemical characterization, a solution of catechol 1 × 10^−3^ M and KCl 0.1 M as the supporting electrolyte was used to test the electrodes’ analytical performance. Electrochemical impedance spectroscopy (EIS) experiments were performed using SOLARTRON impedance analyzer. EIS was used to evaluate the effect of electrocatalytic material on electron transfer resistance. After a stabilization time of 1800 s, the impedance measurements were carried out by applying a signal amplitude of 10 mV, at a working potential of −0.5 V with frequencies varied logarithmically from 0.1 Hz to 100 kHz.

Electrochemical experiments were carried out using a potentiostat/galvanostat PGSTAT128 (Autolab Metrohm, Utrecht, Netherlands). The reference electrode was Ag|AgCl/KCl 3 mol·L^−1^ and the counter electrode was a platinum sheet with a surface of 1 cm^2^. The sensors and biosensors were used as the working electrodes.

Cyclic voltammograms (5 cycles) were registered at a sweep rate of 0.1 V·s^−1^ from −0.6 V to +1.2 V. Four replicas of each sample were measured. The measures on skin extracts and grape seeds were carried out by diluting the samples in KCl 0.1 M. Before each measurement (change of extract), the electrode surface was polished with sandpaper, being tyrosinase deposited again.

### 2.4. Statistical Analysis

The statistical analysis was executed by using Matlab v2020a (The Mathworks Inc., Natick, MA, USA) and R program for statistical computing (version 3.6.2) (The R Foundation for Statistical Computing, Vienna, Austria). PLS was used as a multivariate method to check the perception ability of the said voltammetric e-tongue (electrode array) and to correlate the data with the chemical parameters. The function plsr of the pls library was used in R to obtain PLS regression.

Genetic algorithm is an adequate metaheuristic technique for the data handled in this work with the purpose to verify if variable selection can improve the PLS models performance. The R package “plsVarSel” and its function “ga-pls” (genetic algorithm combined with PLS regression) was used in this procedure. Cyclic voltammograms of the skin and seed extracts were treated separately. For signal processing, the full range of every voltammogram was used. Therefore, the data considered included the oxidation and reduction zones of four repetitions of voltammetric analysis. The number of data was very high (1377 data per cycle) and therefore it was decided to compress the data by Wavelet Data Compression technique [42]. Every cycle was reduced to 22 representative points by means of Haar wavelet. The X (explanatory variables) and Y (responses) data were also centered and scaled by dividing the mean centered data of each variable by its standard deviation. The function plsr of the pls library was used in R to obtain PLS regression.

## 3. Results and Discussion

### 3.1. Phenolic Content: TPC Index and FC Index

Table 1 presents the analytical results obtained for the total polyphenolic and total phenolic contents of grape seeds and skins samples (TPC index and FC index, respectively). As expected, whatever the grape’s variety, seeds showed higher absorbance values than skins, confirming that seeds are the grape components that have the highest content of phenolic compounds. It is known that total extractable phenolics in grape vary between 60–70% in the seeds and 28–35% in the skin, being about 10% or less in the pulp [43].

The seed variability related to the grape variety shows to have an influence on the results of TPC and FC indexes. The phenolic content in seeds is clearly affected by agroecological factors, such as the grape variety, the site of production and the degree of maturation [44,45] being one of the most important in a variety of grapes [46,47,48]. Both indexes agree that Prieto Picudo is the grape variety with highest phenolic content in seeds. On the contrary, the Rufete variety has the lowest levels in both phenolic contents in the seeds.

The skin layer closest to the pulp contains most of the phenolics [49,50] that increase with maturity, whereas they decrease in the seeds [51]; in our case, grape skins of Prieto Picudo variety also showed high content in both indexes; the Tempranillo variety had one of the lowest values in both indices.

### 3.2. Electrochemical Characterization of the Carbon Composite Electrodes Towards Catechol

The objective of this work was to develop a multisensor system based on carbon composite working electrodes with or without sensing materials (referred as EM-electrodes due to the presence of nickel oxide nanoparticles or lutetium phthalocyanine) and tyrosinase enzyme to detect the phenolic content of seeds and skins of grapes. As a first task, the electrochemical characteristics of the three carbon working electrodes (C; C-NiONPs; C-LuPc_2_) towards catechol, a typical phenol present in grapes, was evaluated using electrochemical impedance spectroscopy and cyclic voltammetry.

**Figure 1:**
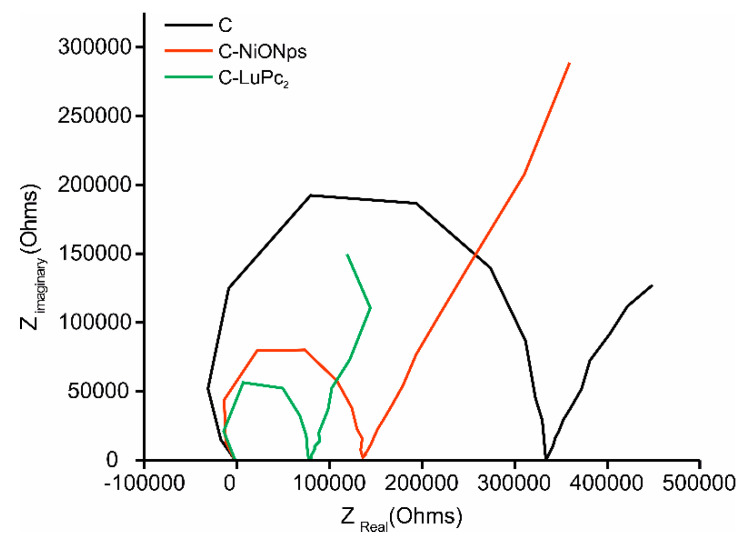
Nyquist plots registered at −0.5 V using (**black**) C (**red**) C-NiONPs and (**green**) C-LuPc_2_ electrodes immersed in catechol 1 × 10^−3^ mol·L^−1^ with 0.1 mol·L^−1^ KCl as the supporting electrolyte. Frequency swept varied logarithmically from 10^−2^ Hz to 10^5^ Hz.

The interfacial electron transfer capabilities of the different electrodes were explored by EIS. Typical Nyquist plots obtained from a 1 × 10^−3^ mol·L^−1^ catechol solution at −0.5 V are displayed in Figure 1. The semicircular part of the diagram at high frequencies corresponds to electron-transfer limited processes and the diameter is equivalent to the electron transfer resistance (R_ct_), which regulates the electron transfer kinetic at the electrode interface. A large diameter was obtained at bare carbon composite electrode (C), indicating that electron transfer process was hindered to a great extent. After the modification of the sensors with electrocatalytic materials, the semicircle diameters decreased sharply. For carbon electrodes modified with metal oxide nanoparticles (C-NiONPs), the semicircle was reduced to half of the bare semicircle, whereas it was reduced to a quarter when modifying the electrodes with bisphthalocyanine (C-LuPc_2_). These results confirmed that the EM were successfully introduced in carbon electrodes.

**Figure 2:**
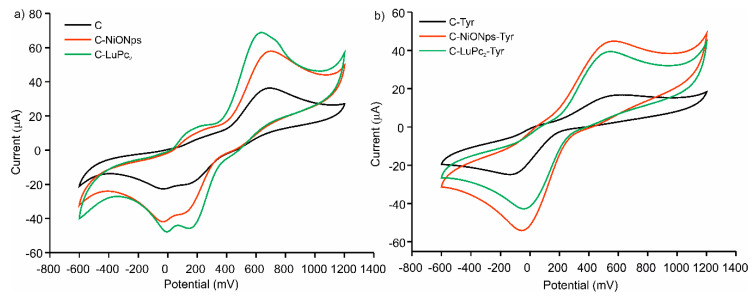
Cyclic voltammograms in catechol 1 × 10^−3^ mol·L^−1^ with 0.1 mol·L^−1^ KCl as the supporting electrolyte. (**a**) C, C-NiONPs, C-LuPc_2_, non-enzymatic electrodes (**b**) C-Tyr, C-NiONPs and C-LuPc_2_-Tyr enzymatic electrodes.

Voltammograms of C, C-NiONPs and C-LuPc_2_ electrodes immersed in catechol 1 × 10^−3^ mol·L^−1^ are shown in Figure 2a. The response was consistent with the expected well-shaped redox pair generated by the two-electron oxidation/reduction of the 1,2-dihydroquinone to 1,2-benzoquinone. In all cases, two oxidation peaks and two reduction peaks were observed, which are associated, according to the literature, to a two-phase mechanism involving two electrons. However, some authors indicate that the appearance of more than one anodic peak is due to the existence of a polymerization process [52,53]. The presence of nanoparticles and phtalocyanines in EM-electrodes caused shifts in the position of the peaks to lower potentials and increases the peaks intensity. The electrocatalytic effect was stronger in C-LuPc_2_ electrode, where an important improvement on the reversibility of the peaks and simultaneously higher intensity currents of the peaks were observed. It can be related to strong π-π interactions between the bis-phthalocyanine and the aromatic structure of catechol that can enhance the electron transfer rate [54,55]. The electrocatalytic activity of NiONPs is associated with their porosity and the mixed valence state found on the nanoparticle surface [56] and it has been already observed in other carbon electrodes [35].

As Tyrosinase is an enzyme selective to the oxidation of o-diphenols, C-EM-Tyr enzymatic electrodes can be also used to detect catechol. In all cases a drastic increase in intensity of the reduction peak was observed (Figure 2b). This increase was due to the reduction of the o-quinone formed by enzymatic oxidation. Also, the important shift of oxidation/reduction potentials observed indicated the improvement in reversibility respect to no-enzymatic electrodes. On the contrary, a single oxidation peak with low current intensity was observed in enzymatic electrodes. This fact had already happened in other biosensors with tyrosinase [57]. The electron transfer was clearly promoted in the presence of electron mediator, such as nanoparticles and/or phthalocyanines. The presence of NiONPs produced the largest increase in the peak intensity (−25 µA in C-Tyr electrode, −42 µA in C-NiONPs-Tyr electrode and −55 µA in C-LuPc_2_-Tyr electrode). The results demonstrated that the electrodes developed in this work facilitated the electron transfer and the synergistic effect between the modifiers and the tyrosinase enzyme.

### 3.3. Discrimination Capability of Grape Extracts with An Array of EM-Carbon Electrodes

The developed electrodes were combined to form an array of sensors that were combined with chemometric methods. The capability of the array of electrochemical electrodes to evaluate the phenolic content of the grapes residues was evaluated by analyzing extracts obtained from skins and seeds prepared from different grape varieties. In all cases, cyclic voltammograms showed a variety of peaks produced by components with redox activity (i.e., phenolic compounds in the 0.4–0.8 V regions).

These voltammograms were characterized by broad peaks whose intensities and positions were determined by the nature of the electrode (enzyme, modifier and substrate) and by the type of the sample. For all varieties of grapes, the peaks were better defined and showed higher intensities for seed extracts in comparison with skin extracts, which is in accordance with the fact that the phenolic content is greater in seeds than in skins, as demonstrated by the TPC and FC indexes.

**Figure 3:**
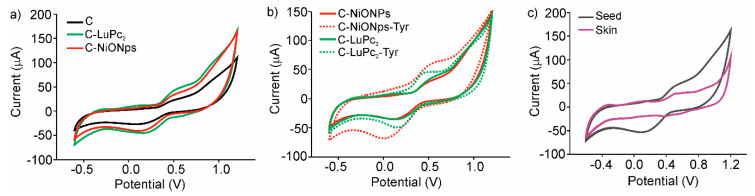
Cyclic voltammograms: (**a**) C, C-NiONPs, C-LuPc_2_, non-enzymatic electrodes applied in the analysis of Cabernet seeds extract (**b**) C-NiONPs, C-NiONPs-Tyr, C-LuPc_2_, C-LuPc_2_-Tyr electrodes applied in the analysis of Prieto Picudo seeds extract (**c**) C-LuPc_2_ electrode applied in the analysis of Garnacha skins and seeds extracts.

Figure 3a illustrates the cross selectivity of the sensors provided by the array of sensors. It is important to remark that all figures correspond to the fifth cycle due to the fact that the first cycle was always different from the rest. However, after five cycles, the signals stabilized and a decrease lower than 5% in the following 15 cycles.

Figure 3b illustrates the notable increase in intensity in the 0.5–0.8 V region (anodic wave) and in the 0.0–0.2 V (catodic wave) when EM-electrodes were used. Results demonstrate the electrocatalytic effect of NiONPs and LuPc_2_ that affects the intensity of the signals related to phenols, although an increase in the peak intensities at negative potentials related to the acidity was also evidenced. As shown in Figure 3b, the enzymatic activity of tyrosinase induces an important degree of selectivity in the electrodes responses, with better defined peaks and higher intensities, especially when electron mediators were used.

Figure 3c illustrates the different responses obtained from skin and seed extracts from different grape varieties. The electrochemical responses of skin extracts were characterized by poorly defined peaks, while the response of seed extracts showed well-defined redox pair potentials with large current densities in the anodic and cathodic waves. The dissimilar responses in skins and seeds extracts were indicative to their different polyphenol content.

**Figure 4:**
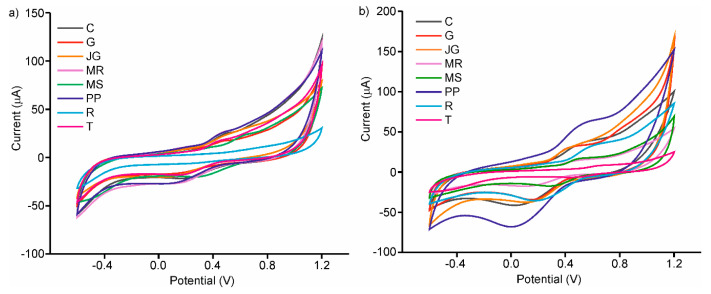
Voltammetric responses of C-LuPc_2_-Tyr enzymatic electrode in (**a**) skins extracts (**b**) seeds extracts of different grape varieties: Cabernet (C), Garnacha (G), Juan García (J), Mencia Regadio (MR), Mencia Secano (MS), Prieto Picudo (P), Rufete (R) and Tempranillo (T).

The peak positions and intensity varied between the various skin extracts (as well as between the various seed extracts), showing a dependence on grape variety. The differences between the voltammograms presented in Figure 4a are linked to the variability in the phenolic composition of the grape skin extracts. The same occurred with the grape seed extracts (Figure 4b). In this case, Prieto Picudo clearly showed the highest intensity peaks, which confirms the highest TPC and FC parameters. That is, as each extract has a different phenolic composition, the oxidation and reduction peaks appear at different potentials and show different intensities. As expected, it is quite evident that the responses for seed extracts showed higher intensities than those obtained in skin extracts. The variety of responses obtained, allowed us to obtain an array of enzymatic electrodes with a high degree of cross selectivity.

### 3.4. E-Tongue: Discrimination Capability and Regression Models to Correlate with Chemical Parameters

PLS was used to obtain a fitting model between the two chemical indexes (TPC and FC indexes, separately) and the input data (wavelets representing the cyclic voltammograms) [58,59]. To validate the results, a full cross validation (leave one out approach) was done; this produced as many calibration sub-models as there were samples in the data set.

**Figure 5:**
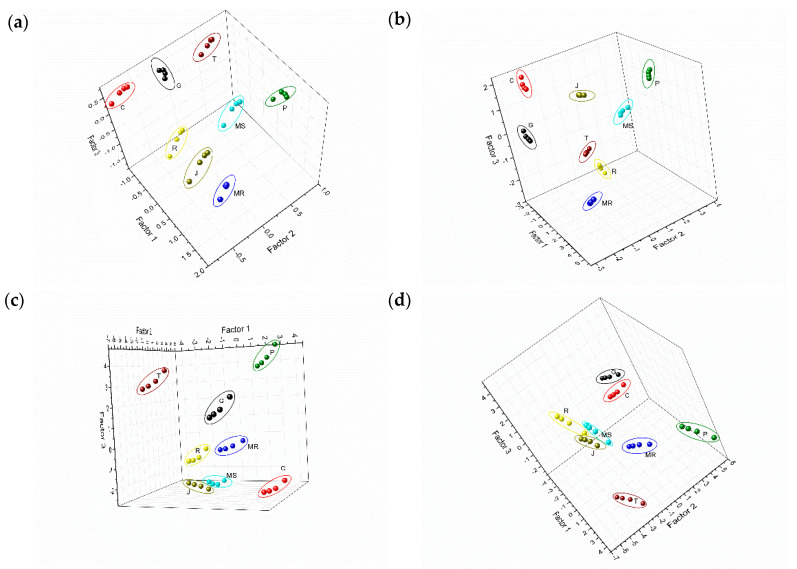
Partial least squares (PLS) score plots of the bio-electronic tongue obtained from voltammetric responses in grape skins extracts, fitted for (**a**) FC index, (**b**) TPC index; voltammetric responses in grape seeds extracts for (**c**) FC index, (**d**) TPC index.

After performing PLS regression using the FC index as response variable, the scores of the three most representative factors for grape skin extracts wavelet CV data are presented in the Figure 5a. It showed well-defined and separated clusters for each grape variety superimposed on a linear surface, showing that all extracts analyzed could be clearly discriminated. In addition, Prieto Picudo (P) skin samples with higher polyphenol index were located in the upper part of the 3D plot toward the right, while the ones with the lowest values are at the bottom of the diagram. It confirms that the levels of phenolic compound play an important role in the discrimination capabilities of the e-tongue. Similar results were obtained from the PLS regression using the FC index as response variable and grape seed extracts wavelet CV data (Figure 5c). As can be observed, the eight seeds extracts are clearly separated and also superimposed on a linear surface showing that the procedure is correct. Clusters were distributed in the plot according to the polyphenolic content (Table 1). Thus, Tempranillo (T) grape seeds (with the lowest values of Folin and TPI) and Prieto Picudo (P) were clearly separated (left and right respectively) from the rest of the extracts. Comparable results can be obtained when applying PLS regression using TPC index as response variable (Figure 5b,d for grape skin and seed extracts wavelet CV data, respectively).

Table 2 presents the overall results (squared correlation coefficient and root mean square error of calibration; squared correlation coefficient and root mean square error of validation; latent variables) of the regression PLS model obtained.

**Figure 6:**
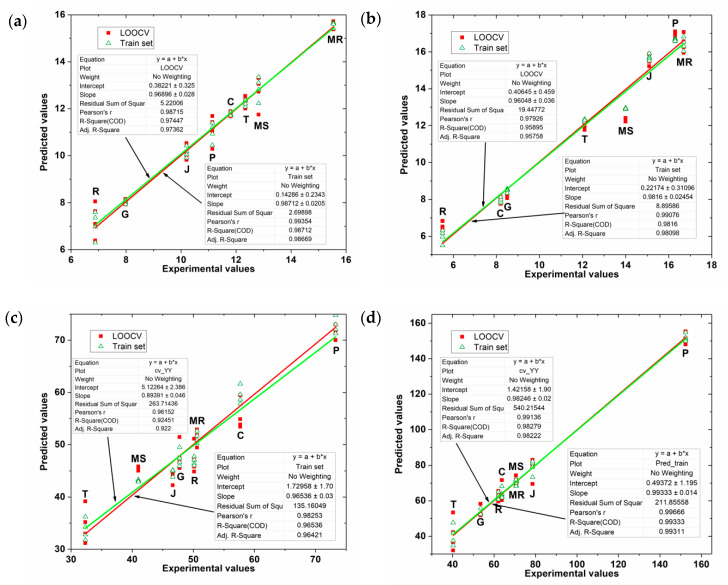
Values predicted by the PLS model for grape skin extracts as a function of the experimental values of (**a**) FC index (**b**) TPC index; and for grape seed extracts as a function of the experimental values of (**c**) FC index (**d**) TPC index.

To assess the quality of the regression PLS model obtained, the FC and TPC indexes predicted by the model using the e-tongue data was correlated with those obtained using the spectrophotometric reference methods (experimental FC and TPC indexes). Figure 6 shows this representation for all the PLS models obtained.

As can be seen in Table 2, PLS model fitting is quite acceptable. The results of the degree of fit of the model in calibration and cross-validation (leave one out approach) are close, having squared correlation coefficient values higher than 0.985, which shows that the obtained PLS models represent more than 98.5% of the damage found in the data. The model’s squared correlation coefficient and error are satisfactory and show that a better PLS adjustment was obtained in the data from the skin extracts than in the seed extracts. Figure 6 shows the linear adjustment obtained with the values predicted by the PLS models as a function of the experimental values of the TPC and FC indices. As can be seen, the data show a linear trend but with high data variability with the exception of the PLS model for grape skin extracts as a function of the experimental values of TPC index. Consequently, these results show that the phenolic compounds indexes in grape skins and seeds can be successfully detected by e-tongue.

In an attempt to improve the PLS models performance, PLS data treatment was repeated together with genetic algorithm (GA) for selecting the best variables within the wavelet CV data. It is recognized that the selection of a reduced set of variables or features can be very beneficial because the model can be greatly simplified and its predictive capabilities improved.

**Figure 7:**
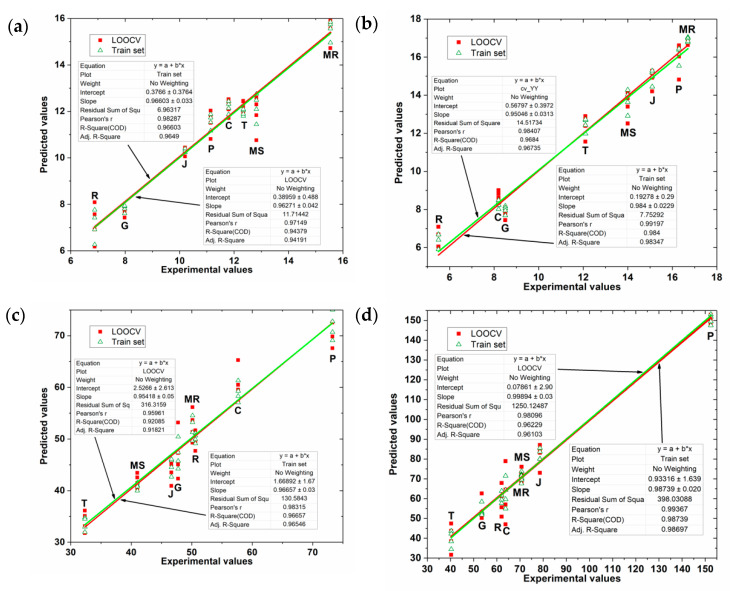
Values predicted by the GA-PLS model for grape skin extracts as a function of the experimental values of (**a**) FC index (**b**) TPC index; and for grape seed extracts as a function of the experimental values of (**c**) FC index (**d**) TPC index.

Figure 7 shows the values predicted by the PLS model for grape skin and seed extracts as a function of the experimental values of TPC and FC indexes. The plots in this figure show a slight reduction in the variability of the points compared to those in Figure 6, showing that the application of the genetic algorithm in wavelet CV data was effective.

Table 3 presents the GA-PLS regression overall results obtained that only in some models there were small improvements. Overall, for grape skin extracts the GA-PLS regression has been able to increase the R2 value and decrease the error values with the same number of latent variables as in conventional PLS regression. In the case of grape seed extracts, it has reduced the number of latent values in a unit by maintaining high values of R2 adjustment and very low error data. However, the regression GA-PLS model to predict TPC values in seed extracts had results inferior than those obtained by the PLS model without applying GA in the selection of variables (Table 2).

## 4. Conclusions

Bio-electronic tongue has been proven to be an effective and reliable tool to relate the voltammetric response of electrochemical modified enzymatic electrodes phenolic extractability parameters in grape seed and grape skin. The performance of this e-tongue dedicated to the analysis of grape residues has been improved using two different strategies. On the one hand, the electrocatalytic properties of the nanoparticles and phthalocyanines as electrochemical modifiers to improve the sensitivity to phenols respect to unmodified sensors. On the other hand, the variable selection method using genetic algorithms (GA) to enhance the statistical analysis. This improved e-tongue is able to discriminate between extracts of seeds and skins from eight different grape varieties. Moreover, good correlations with chemical data, such as total polyphenol content index (TPC index) and total phenolic content index (FC index), were obtained. The bio-electronic tongue shown here combines the advantages of classical electronic tongues with the typical specificity of biosensors. The presence of tyrosinase provides excellent correlations with the polyphenolic content of grape marcs. The system can be used to obtain several parameters in one single experiment.

## Figures and Tables

**Table 1:** Total poliphenol content (TPC) and Folin–Ciocalteu (FC) indexes measured in grape skin and seed extracts (n = 3 and σ ≤ 0.01).

Sample	Extractions	TPC Index	FC Index
**GRAPE SKIN**	Cabernet (C)	12.2	11.8
	Garnacha (G)	8.5	7.98
	Juan García (J)	15.1	10.2
	Mencia Regadio (MR)	16.7	15.5
	Mencia Secano (MS)	14.0	12.8
	Prieto Picudo (P)	21.1	16.3
	Rufete (R)	5.5	6.8
	Tempranillo (T)	12.1	12.3
**GRAPE SEED**	Cabernet (C)	63.8	57.6
	Garnacha (G)	53.5	47.7
	Juan García (J)	78.6	46.6
	Mencia Regadio (MR)	70.7	50.1
	Mencia Secano (MS)	70.6	41.0
	Prieto Picudo (P)	152.4	73.2
	Rufete (R)	62.1	50.6
	Tempranillo (T)	40.3	32.3

**Table 2:** Results of PLS for the skin and seed extracts wavelet CV data and the FC and TPC indexes.

Sample	Parameter	R^2^c ^(a)^	RMSE_C_^(b)^	R^2^_V_^(c)^	RMSE_V_^(d)^	LV ^(e)^
Skin extract	FC index	0.999	0.292	0.999	0.413	5
Seed extract	FC index	0.998	2.09	0.996	3.11	5
Skin extract	TPC index	0.998	0.532	0.996	0.798	4
Seed extract	TPC index	0.999	2.58	0.997	4.15	7

(a) Squared correlation coefficient in calibration; (b) root mean square error of calibration; (c) squared correlation coefficient in validation; (d) root mean square error of validation; (e) latent variables.

**Table 3:** Results of GA-PLS for the skin and seed extracts wavelet CV data and the FC and TPC indexes.

Sample	Parameter	R^2^c ^(a)^	RMSE_C_^(b)^	R^2^_V_^(c)^	RMSE_V_^(d)^	LV ^(e)^
Skin extract	FC index	0.998	0.475	0.994	0.868	4
Seed extract	FC index	0.998	2.06	0.992	4.67	6
Skin extract	TPC index	0.998	0.496	0.993	1.1	4
Seed extract	TPC index	0.998	3.55	0.994	6.25	6

(a) Squared correlation coefficient in calibration; (b) root mean square error of calibration; (c) squared correlation coefficient in validation; (d) root mean square error of validation; (e) latent variables.
